# MOGAD and NMOSD: insights on patients’ radiological and laboratory findings from a single UAE center

**DOI:** 10.3389/fneur.2024.1480723

**Published:** 2024-12-09

**Authors:** Hamdan Alzarooni, Jihad Inshasi, Ahmad Alawadhi, Paul Giacomini

**Affiliations:** ^1^Neurology Department, Rashid Hospital, Dubai, United Arab Emirates; ^2^Department of Neurology and Neurosurgery, Mcgill University, Montreal, QC, Canada

**Keywords:** neuromyelitis optica spectrum disorder (NMOSD), MOGAD, MOG-IgG, United Arab Emirates (UAE), multiple sclerois and neuroimmunology, Dubai, epidemiology

## Abstract

**Introduction:**

Although neuromyelitis optica spectrum disorders (NMOSD) and myelin oligodendrocyte glycoprotein antibody disease (MOGAD) are rare diseases, they pose a significant burden on both society and the healthcare system. This study aims to discuss the demographics and patient characteristics of these diseases in a single center in the United Arab Emirates (UAE).

**Methods:**

This is a retrospective, descriptive study that included patients with either NMOSD or MOGAD treated at Rashid Hospital, UAE during the period between January 2019 and January 2024. Patients were selected and categorized according to NMOSD criteria, aquaporin-4 antibodies, and MOG antibodies. Patient demographics, clinical characteristics, and medical history were retrieved from their medical records and descriptively analyzed in the light of patients’ serological data.

**Results:**

We identified 34 patients with non-multiple sclerosis atypical CNS inflammatory/demyelinating syndromes. Twenty-seven patients (79.4%) fulfilled the criteria for NMOSD, while seven (20.6%) tested positive for MOG antibodies, fulfilling the criteria for MOGAD. In the NMOSD cohort, 19% (*n* = 5) were AQP4-antibody negative. Seventy-four percent of the NMOSD cohort and 43% of the MOGAD cohort were female. For MOGAD patients, disease onset was at a younger age (median onset age of 25 years) compared to the overall study population (mean onset age of 28.94 years). Long segment transverse myelitis was only detected in NMOSD patients (33.3%), and brainstem syndrome with area postrema syndrome was more common in the MOGAD cohort (29% vs. 4%). The rate of positive response to intravenous methylprednisolone as initial therapy was comparable across both cohorts (74% in case of NMOSD and 71% in case of MOGAD).

**Conclusion:**

This study provides valuable insights into the status of NMOSD and MOGAD in the UAE, highlighting the need for larger, prospective studies to further characterize these diseases in the local population, as well as the need for improved understanding of the epidemiology and management of these rare but debilitating conditions.

## Introduction

Neuromyelitis optica spectrum disorders (NMOSD) and myelin oligodendrocyte glycoprotein antibody disease (MOGAD) are both immune-mediated demyelinating disorders frequently involving optic nerve and/or spinal cord causing a wide range of neurological disabilities (1). For decades, patients presenting with the clinical picture now linked to NMOSD (i.e., transverse myelitis [TM], unilateral or bilateral optic neuritis [ON], and brain and spinal cord lesions) were often misdiagnosed with severe multiple sclerosis (MS) (2, 3). Later on, the identification and detection of the antibodies to the protein aquaporin-4 (AQP4-IgG) allowed for the differentiation of these patients from those with MS (2). This has fundamentally transformed the diagnosis and pathogenetic understanding of NMOSD, expanding its defined clinical presentation to comprise cases of acute postrema syndrome (APS) (defined as an episode of otherwise interactable unexplained hiccups or nausea and vomiting), brainstem, hypothalamic, diencephalic and cerebral MRI lesions in addition to the originally described optic neuritis and longitudinally extensive spinal cord disease (2).

Similarly, and following years of their association to NMOSD patients with undetectable AQP4-IgG antibodies (4), the characterization of MOG-IgG antibodies—which was initially detected in 2009 in acute disseminated encephalomyelitis (ADEM) cases (5)—has paved the way for establishing MOGAD as a distinct demyelinating disorder, with its diagnosis centered around a confirmed positive MOG-IgG antibodies test, in addition to the presence of a core clinical demyelinating event (e.g., ON, TM, ADEM, cerebral monofocal or polyfocal deficits, brainstem or cerebellar deficits, and cerebral cortical encephalitis, often with seizures) and the exclusion of MS. Similar to NMOSD, a low positive antibodies test requires further clinical or MRI findings, in addition to a seronegative aquaporin AQP4-IgG test (6).

Despite the similarity in clinical features, NMOSD and MOGAD vary in patient demographics, disease course, prognosis, and treatment outcomes. This can be attributed to the pathogenically different immune-mediation pathways, where the main target of immune attack is myelin in MOGAD versus astrocytes in AQP4 + NMOSD (7). Patients with MOGAD also tend to experience more severe acute attacks compared to those with NMOSD (8, 9). However, MOGAD is also associated with better recovery and less irreversible neurological damage, suggesting more favorable long-term outcomes (10). In contrast, NMOSD, specifically in AQP4 seropositive cases, is marked by a higher risk of accumulated disability over time.

Existing MS treatment options were previously employed for NMOSD patients, primarily due to the overlapping clinical pictures of MS and NMOSD and the absence of medications specific to the latter (11, 12). However, several immunomodulatory drugs that are used in MS were associated with exacerbations in AQP4-ab positive NMOSD patients, which shifted the therapeutic approaches towards selecting medications based on known mechanisms of action for NMOSD and its pathophysiological characteristics (13). Consequently, MS drugs that mainly aim at B-cell depletion were adapted for use in NMOSD, e.g., mitoxantrone, rituximab, and azathioprine (14, 15).

Similarly, therapeutic approaches for MOGAD management have largely been extrapolated from NMOSD management approaches. Yet, emerging evidence indicates that there are distinct differences in how these disorders respond to various treatments (8). This highlights the need for a more tailored, condition-specific approach to managing MOGAD, rather than relying solely on the NMOSD treatment paradigm.

The worldwide estimated prevalence of NMOSD ranges from 0.5 to four per 100,000 and differs greatly among different races and regions (16). Regarding the Arab population, several hospital-based studies that aimed at quantification and characterization of NMOSD patients are published, with one being a retrospective multicenter study in The United Arab Emirates (UAE), which reported a prevalence of 1.76 per 100,000 for NMOSD (17). Additionally, a regional registry for NMOSD in the Arabian Gulf retrospectively reported on 144 NMOSD patients from five Arab countries, including The UAE, with the aim of describing demographical, clinical and radiological findings of NMOSD patients treated in 15 MS and neurology centers in the Arabian Gulf (18). Despite these efforts, data on NMOSD patients in UAE remain scarce. On the other hand, the worldwide prevalence of MOGAD is currently estimated at 1.3–2.5/100,000 (19); however, more accurate estimates are to be anticipated following the recent consensus on MOGAD’s diagnostic criteria. To date, there are no available epidemiological data on MOGAD in the UAE.

Consequently, and given the lack of a national registries for both demyelinating disorders (20), more data on the clinical presentation of NMOSD and MOGAD are needed to quantify their burden and establish standardized diagnosis and management strategies for them. Our study aimed to provide these data on a localized institutional level to precede and call for future generalized initiatives considering larger patient populations.

## Methods

This was a retrospective, descriptive study on all patients having either NMOSD, based on the international NMOSD criteria (2), or MOGAD, identified from cohorts of previously considered seronegative NMOSD, treated and followed in Rashid hospital, a tertiary care center and MS research center in Dubai, UAE. Data collection was initiated on 30^th^ of January 2024, where patients’ data from the preceding five years were included.

Retrieved data comprised patients’ demographics, including age, gender, and ethnicity, as well as disease characteristics including age at onset, disease duration, and attacks presentations; treatments received and patients’ response to them; and laboratory and radiological findings. The collected data was then analyzed in the light of regional and international data.

### Sample size calculation

Given the descriptive nature of our study, we used convenient sampling with no formal sample size calculation. The final sample constituted a total of 34 patients.

### Statistical analysis

This was a descriptive-analysis study. Quantitative variables are presented as mean (standard deviation [SD]) and median (interquartile range [IQR]), and categorical variables are presented as counts and percentages.

### Ethical considerations

The ethics committee approval was obtained from the study site. Anonymity and confidentiality of patients’ data were strictly ensured throughout the study.

## Results

### Patients disposition, demographics, and study cohorts

**Figure 1:**
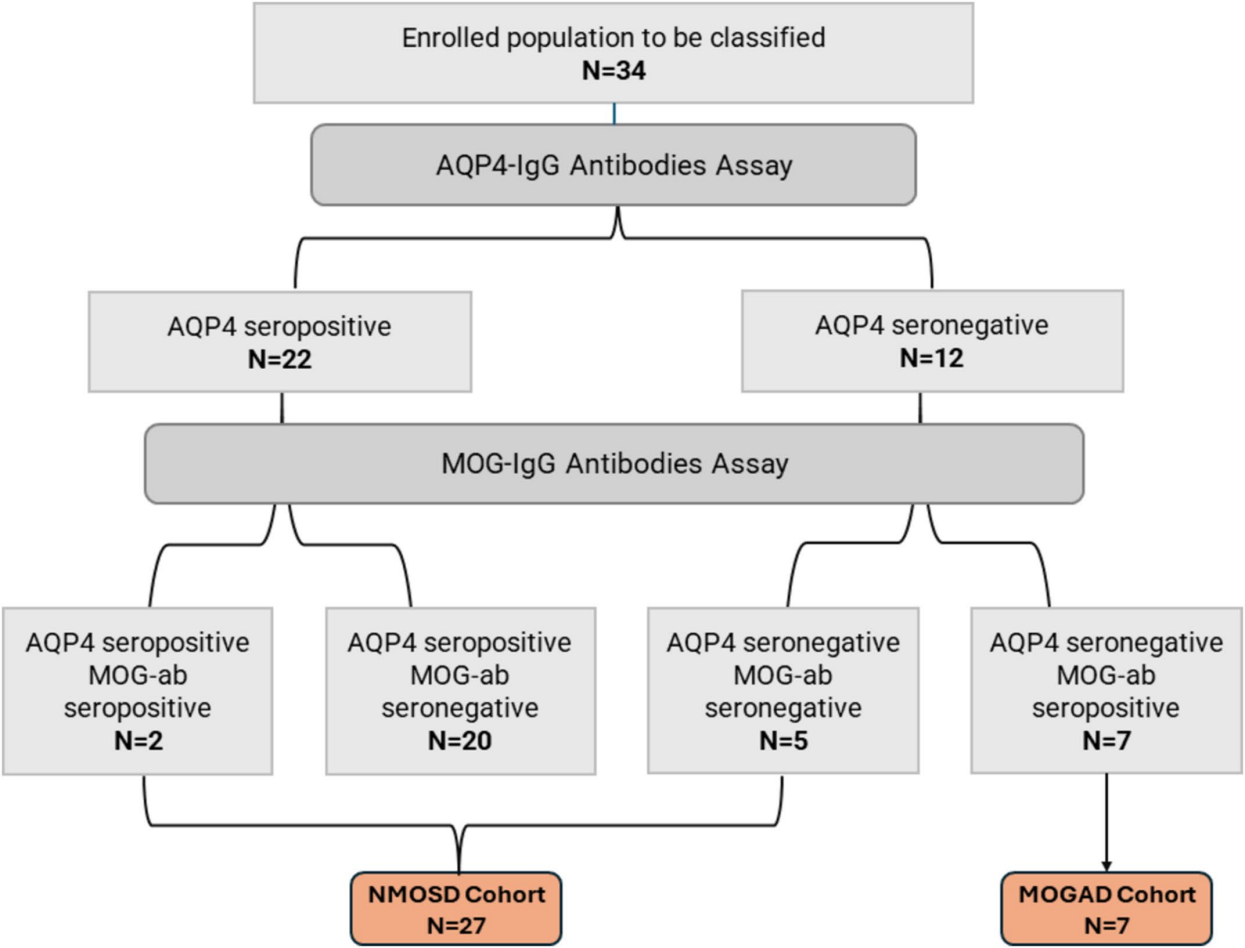
Patients disposition and serological classification.

Thirty-four patients with non-MS demyelinating CNS disorders were included in our study. All patients had undergone testing for AQP4 and MOG antibodies. The detection of AQP4 antibodies was done using bicinchoninic acid assay (BCA) in four patients, enzyme-linked immunosorbent assay (ELISA) in nine patients, and an immunofluorescence assay (IFA) in eight patients. The serology testing method for the rest of the study population was unknown. Patients were classified into two cohorts according to their serological status: (1) NMOSD cohort (*N* = 27), including AQP4 seropositive patients, AQP4 and MOG seronegative patients, and double seropositive patients; (2) MOGAD cohort (*N* = 7), including AQP4 seronegative patients harboring anti-MOG antibodies. More details on both cohorts are illustrated in Figure 1.

Twenty-three of our patients were females, constituting 74% of the NMOSD cohort and 43% of the MOGAD cohort. The majority of the enrolled patients were Arab (73.5%, *n* = 25) and the mean (SD) age at enrollment was 38.1 (13.3) years. More details are available in Table 1.

**Table 1:** Demographics and clinical characteristics of the study population.

Characteristic	NMOSD Cohort, *N* = 27		MOGAD cohort, *N* = 7
	Seronegative, *N* = 5	Seropositive, *N* = 22	
Gender, *n* / *N* (%)			
Female	2 / 5 (40%)	18 / 22 (82%)	3 / 7 (43%)
Male	3 / 5 (60%)	4 / 22 (18%)	4 / 7 (57%)
Ethnicity, *n* / *N* (%)			
African	0 / 5 (0%)	2 / 22 (9.1%)	0/7 (0%)
Arab	5 / 5 (100%)	15 / 22 (68%)	5 / 7 (71%)
Asian	0 / 5 (0%)	2 / 22 (9.1%)	2 / 7 (29%)
Farsi	0 / 5 (0%)	3 / 22 (14%)	0/7 (0%)
Current age—years, mean (SD)	38.1 (13.4)		
	38.6 (15.1)	40.3 (13.1)	29.1 (23.5, 34.1)
Age at onset—years, mean (SD)	28.9 (10.8)		
	31.0 (15.9)	29.2 (9.6)	25.0 (19.5, 31.5)
Disease duration—years, mean (SD)	7.44 (6.4)		
	7.2 (5.4)	9.00 (6.8)	1.0 (1.0, 4.0)
Presentation of attack, *n* / *N* (%)			
Brainstem syndrome	0 / 5 (0%)	4 / 22 (18%)	2 / 7 (29%)
Brainstem syndrome & Area postrema syndrome	0 / 5 (0%)	1 / 22 (4.5%)	2 / 7 (29%)
Long segment transverse myelitis	2 / 5 (40%)	7 / 22 (32%)	0/7 (0%)
Undifferentiated optic neuritis	1 / 5 (20%)	2 / 22 (9.1%)	1 / 7 (14%)
Unilateral optic neuritis	0 / 5 (0%)	4 / 22 (18%)	1 / 7 (14%)
Unilateral optic neuritis & long segment transverse myelitis	2 / 5 (40%)	4 / 22 (18%)	1 / 7 (14%)
Presentation of first attack, *n* / *N* (%)			
Area postrema syndrome	0 / 5 (0%)	1 / 22 (4.5%)	0/7 (0%)
Bilateral optic neuritis	0 / 5 (0%)	0 / 22 (0%)	2 / 7 (29%)
Brainstem syndrome	0 / 5 (0%)	4 / 22 (18%)	2 / 7 (29%)
Long segment transverse myelitis	3 / 5 (60%)	8 / 22 (36%)	1 / 7 (14%)
Optic neuritis & transverse myelitis	0 / 5 (0%)	1 / 22 (4.5%)	0/7 (0%)
Undifferentiated optic neuritis	1 / 5 (20%)	2 / 22 (9.1%)	1 / 7 (14%)
Unilateral optic neuritis	0 / 5 (0%)	6 / 22 (27%)	2 / 7 (29%)
Unilateral optic neuritis & long segment transverse myelitis	1 / 5 (20%)	0 / 22 (0%)	0/7 (0%)
Visual acuity, *n* / *N* (%)			
Blindness	0 / 5 (0%)	1 / 22 (4.5%)	1 / 7 (14%)
Impaired	2 / 5 (40%)	5 / 22 (23%)	3 / 7 (43%)
Normal	3 / 5 (60%)	22 (73%)	3 / 7 (43%)

### Clinical and laboratory data

The mean age of onset for the overall population was 28.94 (10.85), with MOGAD cohort having the lower median age of onset (25.00 [19.5, 31.5]). The first attack presented as long segment TM in 11 NMOSD patients (40%) and only one MOGAD patient (14%), unilateral ON in six NMOSD patients (22.2%) and one MOGAD patient (14%), and brainstem syndrome in four NMOSD patients (12.5%) and 2 MOGAD patients (28.5%). In the case of NMOSD, long segment TM was the most common overall attack presentation (33.3%), while brainstem syndrome and brainstem syndrome with area postrema syndrome corresponded to that in the MOGAD cohort (29% each). Impaired visual acuity was present in 26% of the NMOSD cohort and 43% of the MOGAD cohort. More details on clinical history for each cohort are presented in Table 1.

Cerebrospinal fluid oligoclonal bands were only detected in three patients, all belonging to the NMOSD cohort. MRI findings have shown long spinal lesions in most of the NMOSD cohort (60%) versus only one patient in the MOGAD cohort. However, the MRI findings for the majority of both cohorts did not satisfy McDonald’s criteria. More details on laboratory and radiological findings are available in Table 2.

**Table 2:** Laboratory and radiological findings.

	NMOSD Cohort, *N* = 27		MOGAD Cohort, *N* = 7
Characteristic	Seronegative, *N* = 5	Seropositive, *N* = 22	
Positive cerebrospinal fluid oligoclonal band screen			
Missing	1 / 5 (20%)	4 / 22 (18%)	0/7 (0%)
Negative	2 / 5 (40%)	17 / 22 (77%)	7/7 (100%)
Positive	2 / 5 (40%)	1 / 22 (4.5%)	0/7 (0%)
Long spinal lesions apparent in MRI			
No	0 / 5 (0%)	11 / 22 (50%)	6 / 7 (86%)
Yes	5 / 5 (100%)	11 / 22 (50%)	1 / 7 (14%)
MRI satisfying McDonald’s criteria			
No	3 / 5 (60%)	15 / 22 (68%)	6 / 7 (86%)
Yes	2 / 5 (40%)	7 / 22 (32%)	1 / 7 (14%)

### Medication history

Patients in both cohorts received either intravenous methylprednisolone (IVMP) and/or plasma exchange (PLEX) as an acute therapy for disease attacks. Eighty-five percent of the NMOSD cohort and all the MOGAD cohort initially received IVMP, of which 74 and 71%, respectively, had a positive response to it. Eight NMOSD patients and one MOGAD patient received PLEX, and all of them, except one patient in the NMOSD cohort, reported a positive response to it. More details on both acute and maintenance treatments are presented in Table 3. In both NMOSD and MOGAD cohorts, most of the patients received azathioprine as a first-line maintenance treatment. Overall, four patients experienced side effects that led to first-line treatment discontinuation, while two patients discontinued the first-line therapy due to lack of efficacy: one of which was receiving azathioprine and the other was receiving IVMP. Data on the treatment types and durations are specified in Tables 4, 5.

**Table 3:** Treatments received.

	NMOSD Cohort, *N* = 27			MOGAD Cohort, *N* = 7	
Characteristic	*N*	Seronegative, *N* = 5	Seropositive, *N* = 22	*N*	
IVMP	27			7	
Missing		1 / 5 (20%)	0 / 22 (0%)		0/7 (0%)
No		1 / 5 (20%)	2 / 22 (9.1%)		0/7 (0%)
Yes		3 / 5 (60%)	20 / 22 (91%)		7 / 7 (100%)
Response to IVMP	27			7	
Yes		3 / 5 (60%)	17 / 22 (82%)		5 / 7 (71%)
PLEX	27			7	
NA		1 / 5 (20%)	0 / 22 (0%)		0/7 (0%)
No		2 / 5 (40%)	16 / 22 (73%)		6 / 7 (86%)
Yes		2 / 5 (40%)	6 / 22 (27%)		1 / 7 (14%)
Response to PLEX	27			7	
Yes		2 / 5 (40%)	5 / 22 (23%)		1/7 (14%)
Maintenance treatment^a^	27			6	
AZA		2 / 5 (40%)	12 / 22 (55%)		4 / 6 (67%)
AZA + MMF		0 / 5 (0%)	1 / 22 (4.5%)		0/6 (0%)
AZA + Other		0 / 5 (0%)	1 / 22 (4.5%)		0/6 (0%)
AZA + RTX		2 / 5 (40%)	1 / 22 (4.5%)		0/6 (0%)
MMF		0 / 5 (0%)	1 / 22 (4.5%)		0/6 (0%)
NA		0 / 5 (0%)	1 / 22 (4.5%)		0/6 (0%)
OTHER		1 / 5 (20%)	3 / 22 (14%)		1 / 6 (17%)
RTX		0 / 5 (0%)	1 / 22 (4.5%)		1 / 6 (17%)
RTX + MMF		0 / 5 (0%)	1 / 22 (4.5%)		0/6 (0%)

^a^Treatments are listed in order of treatment setting (i.e., first line followed by second line).

IVMP, intravenous methylprednisolone; PLEX, plasma exchange; AZA, azathioprine; MMF, Mycophenolate mofetil; RTX, rituximab.

**Table 4:** First- and second-line treatments & their durations—NMOSD cohort.

Patient no.^a^	First line	Start	End	Second line	Start	End
1	AZA	2014	2017	MMF	13-Feb-2016	Ongoing
2	AZA	25-Apr-2009	Ongoing	Monthly pulses IVMP	11-May-2013	Ongoing
3	AZA	01-Feb-2017	Ongoing	RTX	30-Oct-2017	Ongoing
4	AZA	01-Feb-2017	2015	RTX	2015	Ongoing
5	MMF	01-Mar-2015	01-Jun-2015	RTX	Nov-2015	Ongoing
6	AZA	23-Oct-2012	Ongoing	–	–	–
7	AZA	01-Jul-2017	Ongoing	–	–	–
8	AZA	01-Mar-2017	Ongoing	–	–	–
9	AZA	2004	Ongoing	–	–	–
10	AZA	20-Nov-2017	Ongoing	–	–	–
11	AZA	2012	Ongoing	–	–	–
12	AZA	2015	Ongoing	–	–	Ongoing
13	AZA	2016	Ongoing	–	–	–
14	MMF	2020	Ongoing	–	–	Ongoing
15	RTX	2020	Ongoing	–	–	–
16	AZA, RTX	2021	Ongoing	RTX	2021	Ongoing
17	Prednisolone, IVIG	2010	2020	IVIG	2020	Ongoing
18	AZA	2019	Ongoing	–	–	Ongoing
19	AZA	01-Nov-2022	Ongoing	–	–	–
20	OFATUMUMAB	24-Jan-2023	Ongoing	–	–	–
21	AZA	01-Jan-2017	Ongoing	–	–	–
22	AZA	20-Jun-2023	ongoing	IVMP	–	–
23	AZA, RTX	Unknown	Ongoing	–	–	–
24	Prednisolone	1-Aug-2023	Ongoing	–	–	–
25	AZA	2008	Ongoing	–	–	–
26	AZA	20-Oct-2023	Ongoing	–	–	–

a Data on maintenance treatment was missing for one participant.

IVMP, intravenous methylprednisolone; PLEX, plasma exchange; AZA, azathioprine; RTX, rituximab; IVIG, intravenous immunoglobulin; MMF, mycophenolate mofetil.

**Table 5:** First- and second-line treatments & their durations—MOGAD cohort.

Patient no.	First line	Start	End	Second line	Start	End
1	AZA	2016	Ongoing	–	–	–
2	RTX	2016	Ongoing	RTX	2016	Ongoing
3	IVMP	2022	–	IVIG	30-Sep-2022	Ongoing
4	IVMP	2022	–	IVIG	10-Aug-2022	Ongoing
5	AZA	2020	2022	IVIG	10-Aug-2022	–
6	AZA	01-Nov-2022	Ongoing	IVMP	7-Oct-2022	–
7	AZA	01-Dec-2022	Ongoing	IVMP	–	–

IVMP, intravenous methylprednisolone; PLEX, plasma exchange; AZA, azathioprine; RTX, rituximab; IVIG, intravenous immunoglobulin.

### Clinical outcomes

#### EDSS median score at onset versus follow up

**Figure 2:**
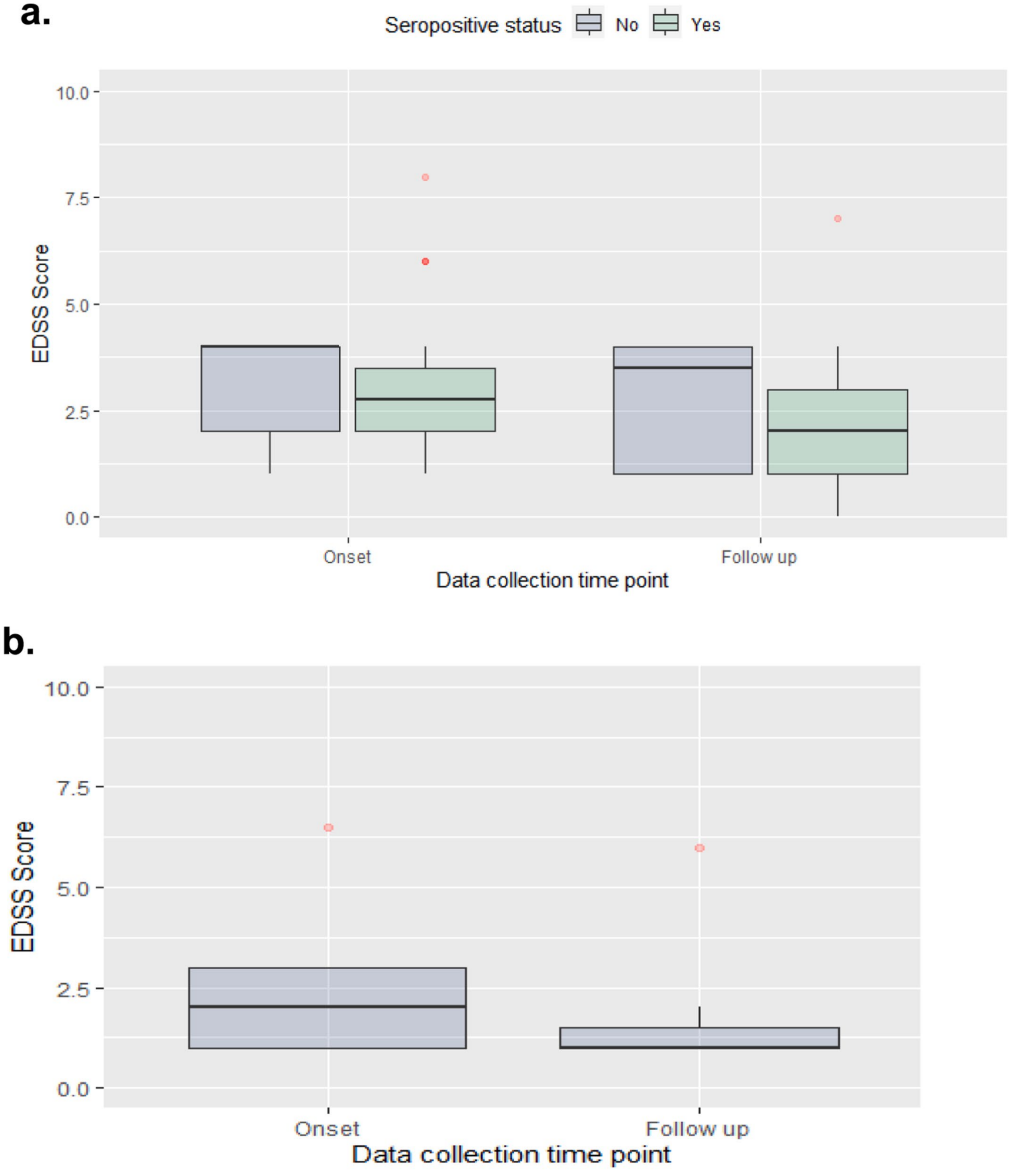
EDSS score at disease onset & last follow-up. (a) NMOSD cohort; (b) MOGAD cohort.

Median (IQR) EDSS scores were reduced from 2.8 (1.5) at disease onset to 2.0 (2.0) at the last follow-up for the NMOSD seropositive group, from 4.0 (2.0) to 3.5 (3.0) for the NMOSD seronegative group, and from 2.0 (2.0) to 1.0 (0.5) in the MOGAD group (Figure 2).

#### Relapses frequency

**Figure 3:**
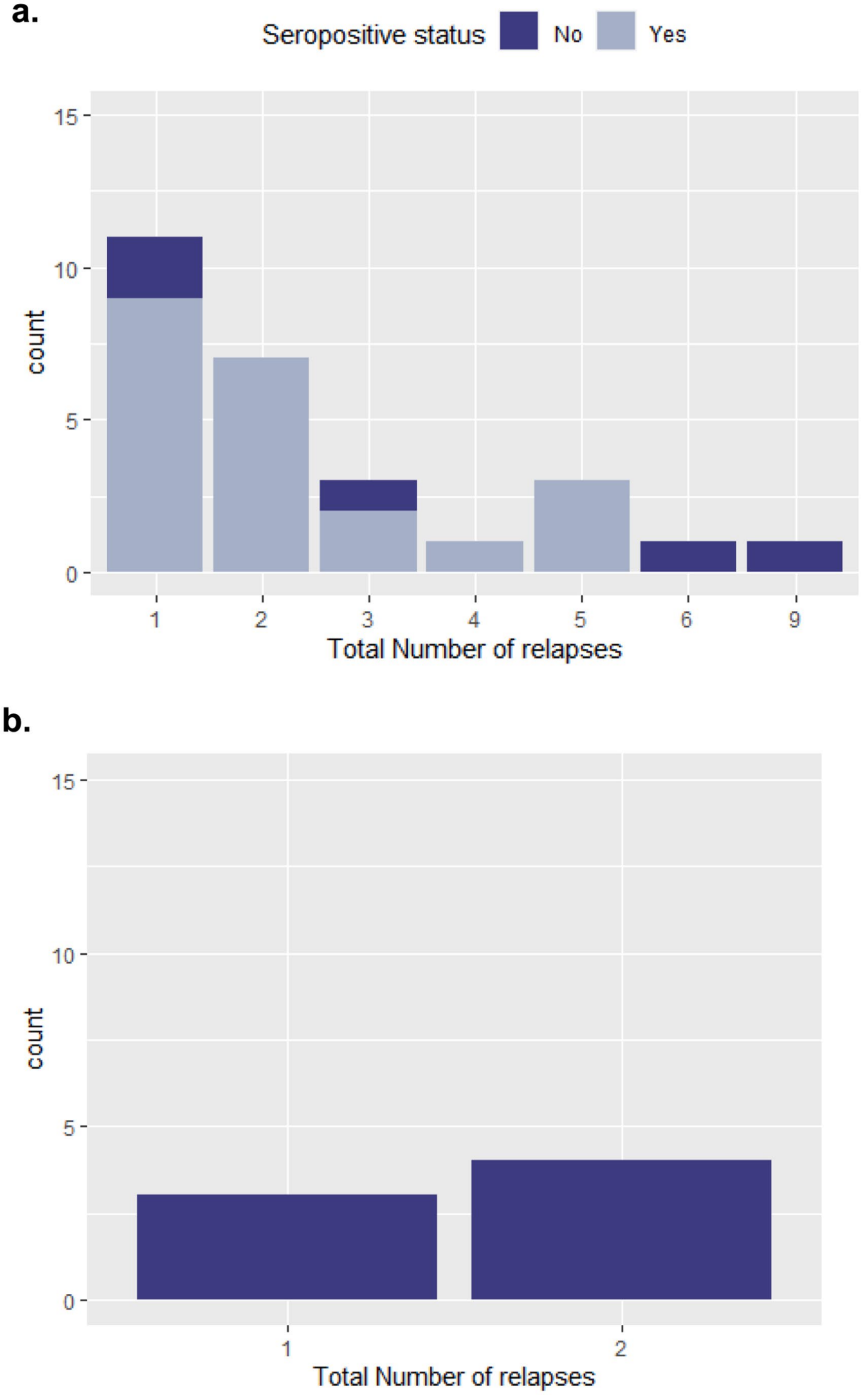
Relapses in the study population. (a) NMOSD cohort; (b) MOGAD cohort.

In the NMOSD cohort, patients experienced up to nine relapses over the follow-up period; however, 40% (*n* = 11) only experienced one relapse during this period. While for MOGAD, the maximum total number of relapses was two, experienced by four of the patients (57%). Five NMOSD patients and three MOGAD patients had at least one relapse in the last year of follow up. More details on number of relapses experienced since disease onset for both cohorts are provided in Figure 3.

## Discussion

Despite the rarity of CNS demyelination diseases, they still represent a great burden on both society and health care system; accordingly, our study discusses the demographics and patient characteristics observed for these diseases in a single UAE center. We identified 34 patients with clinical presentation fitting the clinical picture of non-multiple sclerosis atypical CNS inflammatory/demyelinating syndromes. Twenty-seven patients fulfilled the criteria of NMOSD, while seven patients tested positive for MOG-IgG antibodies fulfilling the criteria for an established MOGAD diagnosis. Regarding the NMOSD cohort, our results match the percentage for AQP4 seronegativity observed for NMOSD in literature, where five patients (19%) were AQP4-ab negative. Previous literature reported that about 20% to 30% of NMOSD patients have undetectable AQP4 antibodies (21). However, 34.7% (*n* = 50) of the 2019 Arabian Gulf NMOSD Registry population, and 53% (*n* = 50) of 103 NMOSD patients included in a population-based study in Iran, were AQP4-ab negative (18, 22). Consequently, and given that many NMOSD cases are still initially misdiagnosed as MS, applying the 2015 NMOSD diagnostic criteria to both AQP4 seropositive and seronegative patients allows for more efficient detection of NMOSD (2, 23).

On the other hand, in parallel with the evolvement of identification patterns for MOGAD, these patients are commonly retrospectively identified in NMOSD patient cohorts with AQP4-ab negative status (24). MOG-IgG antibodies were detected in 11.2% of clinically diagnosed NMOSD seronegative patients in a Chinese study of 125 NMOSD patients (25); similarly, a Brazilian study detected MOG-IgG antibodies in four of 47 patients with AQP4 seronegative NMOSD (26), while an Argentinean study reported that 27.7% of NMOSD seronegative patients tested positive for MOG-IgG antibodies (21). In the Arabian Gulf 2019 NMOSD registry, only 36% (*n* = 18) of the AQP4-seronegative patients were tested for MOG-IgG antibodies, with no positive results returned (18).

Two patients in our study cohort were double positive for both MOG-IgG and AQP4-IgG antibodies. This status was previously reported in an individual-case manner (4, 25, 27, 28); however, whether this population requires distinct management strategies is yet unknown. Similar to previous studies, the sample size in our study was too low to evaluate any of these outcomes in this population.

In our study, we compared patients with a definite NMOSD diagnosis to those with MOG antibodies—who were initially considered for an NMOSD diagnosis. For instance, the female predominance observed for NMOSD, particularly AQP4 seropositive cases, was not mirrored in the MOGAD cohort. Similar trend was observed in previous trials (8, 29, 30), where the female-to-male ratio can reach up to 3–9:1 in NMOSD, in contrary to a ratio of 1:1 in MOGAD (29, 31). Regarding the age of onset, our results go in line with literature suggesting that MOGAD usually present at a younger age than that for NMOSD (4). In contrast, in a study of 215 patients comparing NMOSD and MOGAD characteristics, the median onset age reported for MOGAD patients was 37.5 years, and that for APQ4 seropositive NMOSD was 37 years, while the lowest age of onset (32.5 years) was reported for the APQ4 seronegative study population (30). In a population-based study in Tehran, the mean age of onset was 31.5 years (range 8–61), which also matched that of the Arabian Gulf registry (31 ± 12 years) (18, 22).

In our cohort, the immunosuppressive maintenance therapies included azathioprine, IVMP, IVIG, mycophenolate mofetil (MMF), or rituximab as initial therapy, with a combination of the first and the latter in two NMOSD patients, and the combination of prednisolone and IVIG in another. In non-MS demyelinating disorders, treatment outcomes highly vary with treatment duration and disease onset and clinical picture. For instance, rituximab’s efficacy correlates with B cells levels, which are also subject to individual variations (32, 33). This calls for extensive understanding the relationships between patient variables and treatment outcomes; however, the small sample size in our study hindered the quantifications of such outcomes to drive the needed conclusions.

Regarding recovery degrees, reflected by the change in EDSS following the follow up period, MOGAD patients had greater median EDSS change and lower median EDSS scores at follow-up, which goes in line with previously reported data for MOGAD (34). For NMOSD, the seropositive group has demonstrated a slightly higher median EDSS change (0.8 vs. 0.5), which may be attributed to the lower baseline EDSS (2.8 vs. 4.0) (35). In literature, improvement in mean EDSS is comparable between seropositive and seronegative NMOSD populations (18).

In our study, most of the NMOSD AQP4 positive patients (82%) reported a positive response to the first line acute therapy IVMP; the same outcome was observed in the MOGAD cohort. Azathioprine was the most common maintenance therapy in both cohorts; however, our cohort of patients received different treatments with different durations, which hinders the interpretation of treatment outcomes in the light of other patient characteristics. This also may explain the variation observed in the number of relapses experienced by the study patients.

Our study has the limitation of small sample size, especially in the MOGAD cohort; therefore, care must be employed upon deriving definite conclusions where generalization is not optimal given the convenient sampling approach. However, our study lays the ground for further research to evaluate these outcomes in adequately powered comparative studies. Due to the retrospective nature of this study, correlation assessments for different clinical features, disease course, and the response to immunotherapies were not done. In that context, our study also highlights the limitations in logistical capabilities which complicated reporting on a larger array of disease features. Moreover, our study was conducted on an institutional level, while population-based studies are needed to assess the prevalence and incidence rates. However, conducting such studies for conditions like NMOSD and MOGAD may be challenging. This is because it can be difficult to establish consistent diagnostic criteria across different settings and to standardize the laboratory testing for the associated antibodies. Without these unifying factors, accurately assessing the true population-level epidemiology of NMOSD might not be feasible.

## Conclusion

Our study provided valuable insights on the status of both NMOSD and MOGAD in our center; however, adequately powered prospective studies applying standardized definitions and methodologies are needed to further characterize NMOSD patients clinically and epidemiologically in the UAE.

## Data Availability

The raw data supporting the conclusions of this article will be made available by the authors, without undue reservation.
